# Ostomy Does Not Lead to Worse Outcomes After Bowel Resection With Ovarian Cancer: A Systematic Review

**DOI:** 10.3389/fonc.2022.892376

**Published:** 2022-05-23

**Authors:** Xinlin He, Zhengyu Li

**Affiliations:** ^1^ Department of Gynecology and Obstetrics, West China Second University Hospital, Sichuan University, Chengdu, China; ^2^ Department of Obstetrics and Gynecology, Key Laboratory of Birth Defects and Related Diseases of Women and Children, Ministry of Education, West China Second University Hospital, Sichuan University, Chengdu, China

**Keywords:** ostomy, ovarian cancer, bowel resection, meta-analysis, systematic review

## Abstract

**Background:**

Debulking cytoreduction surgery with bowel resection is a common intervention for ovarian cancer. It is controversial whether ostomy causes worse survival outcomes and how clinical physicians should choose which patients to undergo ostomy. During this study, we performed a systematic review to determine whether ostomy leads to worse outcomes after bowel resection compared to anastomosis. We also summarized the possible indications for ostomy.

**Methods:**

We searched PubMed, Embase, and Cochrane for articles containing the phrase “ovarian cancer with bowel resection” that were published between 2016 and 2021. We included studies that compared primary anastomosis with ostomy. We mainly focused on differences in the anastomotic leakage rate, length of hospital stay, overall survival, and other survival outcomes associated with the two procedures.

**Results and Conclusion:**

Of the 763 studies, three were ultimately included in the systematic review (N=1411). We found that ostomy did not contribute to worse survival outcomes, and that the stoma-related complications were acceptable. Indications for ostomy require further study. Bowel resection segment margins and the distance from the anastomosis to the anal verge require consideration.

## Introduction

Bowel metastasis frequently occurs with advanced ovarian cancer, and debulking cytoreduction surgery, especially en bloc cytoreduction, is recommended (1–3). Bowel resection is performed when bowel metastasis is observed before or during surgery, and it is followed by primary anastomosis or ostomy (4–7). Primary anastomosis is the first choice after bowel resection, and ostomy is the alternative choice. Ostomy is not preferred because it has been shown to cause a worsened quality of life with ovarian cancer (8, 9).

Ostomy is the creation of an artificial anus in the abdomen. Because ostomy can be reversed, it can be permanent or temporary. For ovarian cancer patients, ostomy is performed to divert the feces so that the anastomosis can recover well. Anastomotic leakage (AL) is one of the most important complications associated with anastomosis because it can cause abdominal inflammation and some other issues (10). After bowel resection, ostomy can be performed instead of primary anastomosis to prevent AL. Diverting ileostomy is one of the common choices with ostomy because it is believed that it can decrease the AL rate associated with colorectal cancer (11). The AL rate has not shown any difference with permanent ostomy and temporary ostomy for colorectal cancer (12). However, patients with a stoma experience complications such as skin irritation and prefer to avoid ostomy to ensure a better quality of life (13–15). Physicians need to evaluate the problems and benefits before deciding whether to perform ostomy.

No guidelines specifically recommend follow-up interventions after bowel resection in ovarian cancer patients (1, 16, 17). Clinical physicians make decisions based on experience. There is no clear answer regarding whether ostomy will benefit patients with ovarian cancer. Few studies have concentrated on bowel surgery and its outcomes when ostomy and anastomosis are performed for ovarian cancer patients. It is still controversial whether ostomy causes worse survival outcomes and how clinical physicians should choose which patients to undergo ostomy. This review analyzed studies performed during the past 5 years to determine whether ostomy leads to worse outcomes than anastomosis. The possible indications for ostomy are also summarized.

## Materials and Methods

**Figure 1:**
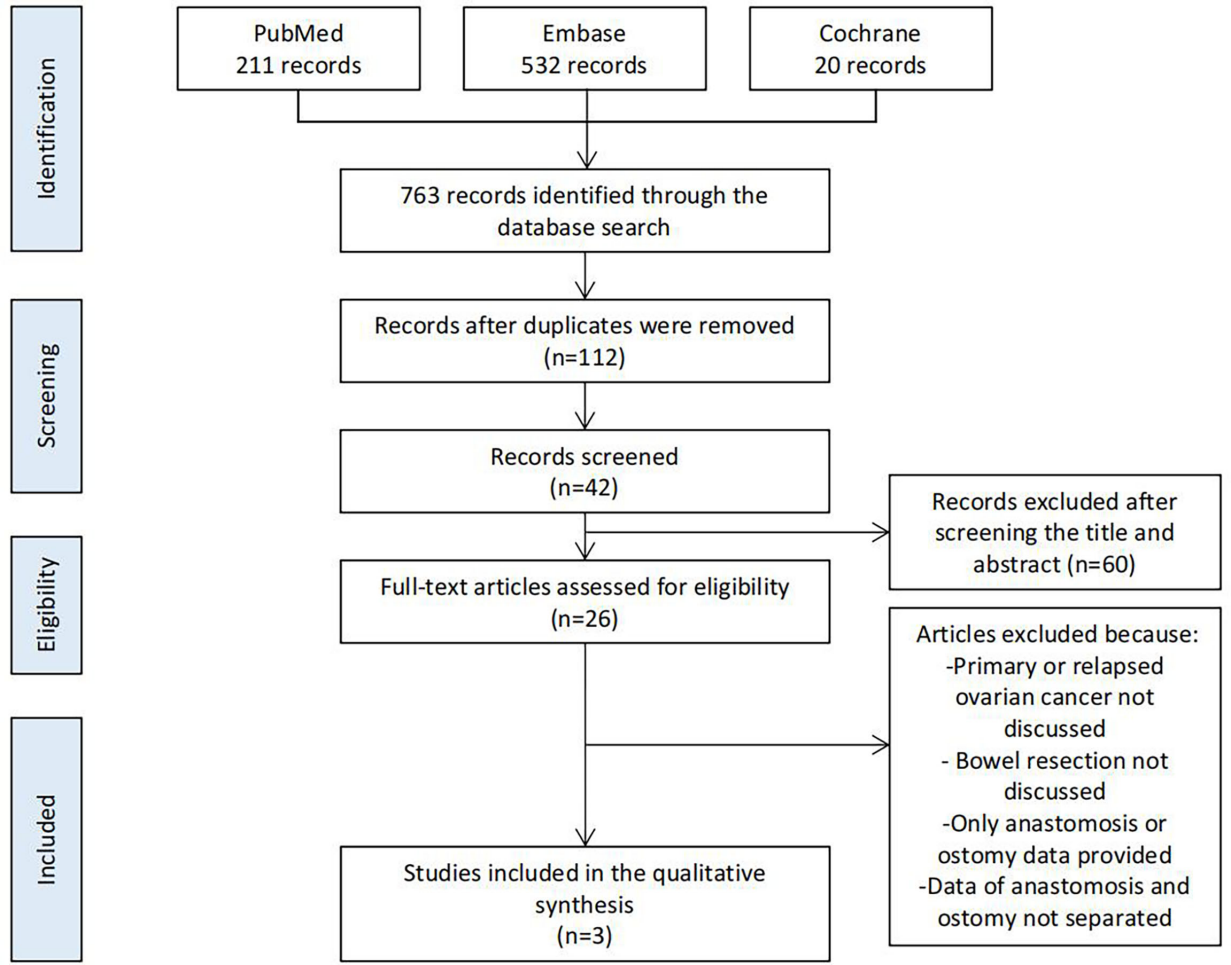
PRISMA diagram.

We searched PubMed, Embase, and Cochrane using the following MeSH terms and keywords in articles published during the past 5 years: [ovarian cancer] AND [ostomy] OR [anastomosis]. After excluding repeated studies, we screened all articles based on the title, abstract, and full text (**Figure 1**).

According to the PICO principle, P was primary or relapsed ovarian cancer, I was ostomy after bowel resection, and C was primary anastomosis after bowel resection. All meta-analyses were required to meet the following criteria: patients had primary or relapsed ovarian cancer; all patients underwent bowel resection during primary debulking surgery or interval debulking surgery, and data of ostomy patients were separated from those of anastomosis patients.

Age, body mass index, American Society of Anesthesiology score, and medical history were recorded as baseline data. Surgical information was recorded as a variable. We mainly focused on short-term and long-term outcomes such as the anastomotic leakage (AL) rate, length of hospital stay, 30-day readmission, overall survival (OS), progression-free survival (PFS), and other survival outcomes. This systematic review was not registered.

## Results

Of the 763 studies found during the database search, 112 remained after screening for duplicates. After screening the title, abstract, and full text based on the criteria, three studies were finally analyzed. Twenty-two other studies were used as references to support our interpretations.

### Major Findings of the Three Studies

Three retrospective studies directly compared the outcomes of ostomy and primary anastomosis. These were the most important references during our analysis (**Figure 1** and **Table 1**).

**Table 1:** Information collected from the three studies.

	Canlorbe et al.				Fleetwood et al.				Lago et al.		
	Primary anastomosis		Ostomy		Primary anastomosis		Ostomy		Primary anastomosis / Ghost ileostomy		Ostomy
**Basic information**											
Age (mean)	53		53		60.1 (overall)				55.7 / 54.5		60.9
BMI (mean)	24		23		Not reported				25.1 / 24.8		25.1
ASA score											
1	7.8%		0		Not reported				81.9% / 92.9% (combined scores 1 and 2)		63.2% (combined scores 1 and 2)
2	67.8%		55.6%								
3	18.9%		44.4%								
Unknown	5.6%		0								
Comorbidities					86.3%		85.9%				
Hypertension	18.9%		3.3%		41.4%		40.6%		Not reported		
Diabetes	12.2%		11.1%		Not reported				2.8% / 2.4%		10.5%
Corticoid/steroid use	1.1%		0		3.1%		3.6%		2.8% / 7.1%		0
FIGO stage											
Not applicable (relapse)					Not reported				8.3% / 9.5%		10.5%
II									6.9% / 9.5%		0
III	92.2%		100%						59.7% / 61.9%		84.2%
IV	7.8%		0						25.0% / 19.0%		15.8%
Preoperative serum results											
CA125 (>500 U/mL)	35.6%		22.2%		Not reported				Not reported		
Hyperalbuminemia (<3.5 g/dL)	Not reported								25.0% / 23.8%		10.5%
Mean albumin level (g/dl)					3.73		3.58				
Compared the baseline data between groups	Yes				Yes				Yes		
											
**Surgery information**											
Mean operative time (min)	466		557		Not reported				363 / 360		402
Intraoperative transfusion	Not reported								61.1% / 42.9%		78.9%
Mean intraoperative blood loss (mL)	1508		1100						446 / 557		683
Repeat bowel resection	32.2%		66.7%						18.1% / 14.3%		31.6%
Additional surgery											
Lymphadenectomy	85.6%		66.7%						55.6% / 69.0%		47.4%
Omentectomy	Not reported								90.3% / 88.1%		84.2%
Low anastomosis from the anal verge (<5 cm)	Not reported								6.9% / 14.3%		5.3%
Compared the surgery information	Yes				No				Yes		
											
**Outcomes**											
Mean hospital stay (day)	18		20		Not reported				10 (ghost ileostomy)		11
Adverse events (grade III or higher)	27.8%		22.2%		36.5%		36.9%				
Stoma-related adverse event									7.1% (ghost ileostomy)		78.9%
AL rate	6.7%(in Grade III)		11.1%(in Grade III)		Not reported				5.6% / 4.8%		5.3%
Reverse rate			88.9%		Not reported						73.7%
Median overall survival	31 months								Not reported		
Progression-free survival	17 months										
30-day morbidity					Ostomy status: OR, 1.97 (univariate)						

AL, anastomotic leakage; ASA, American Society of Anesthesiology; BMI, body mass index; FIGO, International Federation of Gynecology and Obstetrics; OR, odds ratio.

The study by Canlorbe et al. included stage IIIB to IV ovarian cancer based on the International Federation of Gynecology and Obstetrics (FIGO) classification with anterior bowel resection during complete cytoreductive surgery (18). Patients were divided into groups based on whether they underwent ileostomy/colostomy (without stoma group, N=90; with stoma group, N=9). Of the nine patients with a stoma, one patient had two stomas and one was scheduled to undergo left colostomy but it was changed to ileostomy. Some baseline data, such as age and BMI, were reported. They also reported some surgery information, including the number of stomas and whether small bowel resection was performed. They compared the basic data and surgery information and found no difference between groups. A reverse rate of 88.9% and overall AL rate of 7.1% (6.7% in the primary anastomosis group and 11.1% in stoma group; not statistically compared) were reported during this study. Three adverse events (lower than Clavien–Dindo grade III) caused by the stoma were reported. The median OS was 31 months and the median PFS was 17 months. During the univariate analysis, patients with a stoma had a longer hospital stay and worse OS and PFS (log-rank test). The multivariate analysis was performed after the univariate analysis. Ileostomy and lymph node involvement were found to be risk factors for relapse. They reported two OS and PFS curves, but these were not statistically compared. This retrospective study reported worse outcomes for the stoma group; however, it did not report multivariate analysis results regarding survival outcomes and did not compare the curves of the two groups using multivariate statistics. The sample size was small, especially that of the stoma group, which made the results less reliable. The association between the surgery type and the outcomes was not reported.

The study by Fleetwood et al. included ovarian cancer patients who underwent colon resection. These patients were divided into the primary anastomosis group (N=453) and end ostomy group (N=586) (19). Some basic information was reported and the preoperative comorbidities were compared between groups (no significant difference). The primary anastomosis group seemed to have significantly more disseminated cancer, and the stoma group experienced more preoperative weight loss and received more neoadjuvant chemotherapy; however, the differences were not significant. Lower preoperative albumin and platelet levels and significantly higher preoperative leukocyte counts were observed in the ostomy group. Surgery information was not recorded in detail. Postoperative complications were almost statistically equal between groups, but the ostomy group tended to have worse complications. Severe adverse events (Clavien–Dindo grades III and IV) were equal between groups. However, the ostomy group had more grade II adverse events. The 30-day mortality rate was higher in the ostomy group in this study (3.1% in the primary anastomosis group and 6.2% in the stoma group). However, ostomy was not an independent risk factor when the preoperative laboratory values were controlled in the logistic regression. The blood urea nitrogen, creatinine, and preoperative albumin levels contributed to death. This retrospective study reported worse outcomes for the stoma group; however, the results changed after performing the multivariate analysis. The baseline data were compared and multivariate analysis was performed, which made these study results more reliable. However, surgery information was not recorded in detail, making the study results less reliable. It is unknown whether equal numbers of surgery types were performed. Furthermore, the reversal rate and AL rate were not reported.

The study by Lago et al. included patients with FIGO stages II to IV (20). These patients underwent colorectal resection after anastomosis and were divided into three groups (wait and see group, N=72; diverting ileostomy group, N=19; ghost ileostomy group, N=42). Ghost ileostomy involved the cutaneous placement of a portion of the terminal ileum. If no AL was observed by 7 days after surgery, then the ileum was reversed. If any AL occurred, then an ileum incision was performed without repeat laparotomy. Colonoscopy at 3 days and colonoscopy at 7 days after surgery was performed to find the AL. Some baseline information, including the albumin level, was recorded. There were no statistical differences among the three groups. Surgery information was recorded in detail. Statistical differences were observed in the estimated blood loss and intraoperative transfusion rate among groups (both were higher in the ileostomy group). The AL rate was equal among the three groups (5.6% in the primary anastomosis group; 5.3% in the ileostomy group; 4.8% in the ghost ileostomy group). The ileostomy group had a reversal rate of 73.7%; however, the ghost ileostomy group had a reversal rate of 100%. The median hospital stay and the interval between surgery and chemotherapy were not different between the ileostomy group and the ghost ileostomy group. The ileostomy group had a higher rate of stoma-related complications than the ghost ileostomy group. This retrospective study compared the baseline data and surgery information among three groups, thus making its results more reliable. Colonoscopy was beneficial for finding asymptomatic AL and resulted in a more reliable AL rate. However, no multivariate analysis was performed and the sample size was small, thus making the results less reliable. Survival outcomes were not reported by this study.

These three studies reported different AL rates and survival outcomes for the primary anastomosis group and ostomy group. To further analyze these three studies, we used other studies as a reference.

### Interpretation of the Major Finds

Higher AL rates contributed to worse OS (21). Hypoalbuminemia was an independent risk factor for AL, as reported by many studies (21–24). In fact, clinical physicians believed that the albumin level indicated whether ostomy should be performed (18–20). It was suggested that preoperative hypoalbuminemia might contribute to worse OS. According to the studies by Canlorbe et al. and Fleetwood et al., OS was worse for the ostomy group (18, 19). However, the study by Canlorbe et al. did not mention the albumin levels of the groups (18). The study by Fleetwood et al., after controlling the albumin level, reported no difference in the OS of the primary anastomosis group and ostomy group (19). The study by Lago et al. also reported no differences in the AL rates and albumin levels of the two groups; however, the survival outcomes were not directly compared (20). Therefore, ostomy itself would not contribute to worse OS with ovarian cancer.

The study by Canlorbe et al. reported that ileostomy and lymph node involvement were risk factors for relapse and observed higher PFS in the ostomy group (18). Gallotta et al. performed a multivariate analysis and indicated that metastatic mesenteric lymph nodes were associated with high rates of isolated aortic and celiac trunk lymph node recurrences (25). However, we did not find any other studies that supported ostomy as an independent risk factor for relapse. Canlorbe et al. explained that patients with a stoma received fewer cycles of adjuvant chemotherapy because of poorer compliance (18). However, their small sample size and lack of a multivariate analysis made the results less reliable.

Many stoma-related complications, such as dehydration and malnutrition, decrease the quality of life (26). Many patients do not prefer ostomy because it is associated with a worse quality of life. However, stoma-related complications rarely cause Clavien–Dindo grade III or higher adverse events with ovarian cancer, which is acceptable (18–20). It has been reported that the reverse rate varies from 43.3% to 88.9% (18, 20, 27). Enhancing postoperative care and increasing the reverse rate might result in patients being more receptive to ostomy.

We summarized and analyzed three studies that directly compared primary anastomosis and ostomy. We found that ostomy alone did not contribute to worse survival outcomes and that the stoma-related complications were acceptable.

### Indications for Ostomy

Ostomy, especially diverting ileostomy, is believed to reduce the AL rate and may result in better survival outcomes; however, these results were not reported by the three aforementioned studies (18–20). The indications for ostomy might contribute to the AL rate, thereby rendering the difference between primary anastomosis and ostomy not significant. A decreased AL rate was reported for ostomy by Kalogera et al., who compared the AL rate before and after determining the indications for ostomy (26).

For ovarian cancer, there are no guidelines that specifically recommend ostomy after bowel resection (1, 14, 15). Indications causing physicians to perform ostomy have been partially different from the real risk factors for AL (28). Some studies have reported that hypoalbuminemia (<3.5 g/dL), additional bowel resection, more extensive rectosigmoid resection, previous treatment with bevacizumab, longer operative time, and intraoperative red blood transfusion might lead physicians to perform ostomy (27–29). However, in other studies of the risk factors for AL, age, preoperative albumin level, small intestine resection, positive resection margins, additional bowel resection, manual anastomosis, and the distance from the anastomosis to the anal verge were independent risk factors for AL (21–23, 30–33). Clinical physicians chose patients with a worse preoperative status, such as malnutrition and elderly age, to undergo ostomy, which were proven to be risk factors for AL (22, 28). Regarding the surgery information, additional bowel resection and additional small intestine resection were proven to be risk factors for AL and were evaluated by physicians to decide whether to perform ostomy (21, 22, 27, 31, 32). However, physicians in the gynecology field might ignore some surgical factors, such as bowel resection margins and the distance from the anastomosis to the anal verge. Furthermore, risk factors for AL were not unanimous in all of these studies; different potential factors were included in the aforementioned studies. It should be proven whether risk factors for AL affect outcomes of ovarian cancer by directly comparing them using multivariate analysis.

No clear indications for ostomy have been suggested by the guidelines for ovarian cancer. We suggest that clinical physicians should evaluate the preoperative status and surgery information of patients before deciding whether to perform ostomy. The preoperative albumin level and age were the most important preoperative characteristics. However, additional bowel resection, additional small bowel resection, bowel resection margins, and the distance from the anastomosis should be considered. Furthermore, a multivariate analysis of all possible indications is needed to prove the conclusions of these studies.

## Discussion

The aforementioned studies did not allow us to perform a meta-analysis to obtain a more precise and statistical conclusion. We were only able to review the literature and report our suggestions.

Ostomy itself did not contribute to worse outcomes, and the AL rate decreased after considering the indications for ostomy (26). Further investigations of the indications for ostomy with ovarian cancer are needed. Because the AL rate is reportedly low for ovarian cancer (approximately 5%), larger sample sizes are needed for future studies. Some studies of the risk factors for AL showed that postoperative colonoscopy might be useful for identifying asymptomatic AL (34–36) after surgery. Moreover, studies of colorectal cancer might suggest some factors that could affect decision-making when ovarian cancer is involved. For example, the distance from the tumor to the anal verge was associated with the AL rate and survival outcomes of colon cancer; this has been proven but is not usually considered an indication by physicians treating ovarian cancer (23, 37).

## Conclusion

Ostomy did not contribute to worse survival outcomes after bowel resection compared with primary anastomosis with ovarian cancer. The stoma-related complications were considered acceptable. The basic characteristics of patients (age and preoperative albumin level) and surgery information (operative time, intestine resection, additional bowel resection, manual anastomosis, bowel resection margins, and the distance from the anastomosis to the anal verge) should be considered before performing ostomy and require further investigation.

## Data Availability Statement

The raw data supporting the conclusions of this article will be made available by the authors, without undue reservation.

## Author Contributions

XH searched the database and did the statistic analysis, as well as wrote this manuscript. ZY provided practical suggestions and critically revised the manuscript. All authors contributed to the article and approved the submitted version.

## Funding

This study was funded by the Sichuan Province Science and Technology Support Program (grant number 2019YJ0072).

## Conflict of Interest

The authors declare that the research was conducted in the absence of any commercial or financial relationships that could be construed as a potential conflict of interest.

## Publisher’s Note

All claims expressed in this article are solely those of the authors and do not necessarily represent those of their affiliated organizations, or those of the publisher, the editors and the reviewers. Any product that may be evaluated in this article, or claim that may be made by its manufacturer, is not guaranteed or endorsed by the publisher.

## References

[1] Purandare CN . FIGO Cancer Report. Preface. Int J Gynaecol Obstet (2018) 143(suppl. 2):1. doi: 10.1002/ijgo.12607 30306587

[2] Sebastian A Thomas A Varghese G Yadav B Chandy R Peedicayil A . Outcome of Bowel Resection in Women With Advanced Ovarian Carcinoma. Indian J Surg Oncol (2018) 9:511–8. doi: 10.1007/s13193-018-0790-2 PMC6265198 30538381

[3] Son JH Kong TW Paek J Chang SJ Ryu HS . Perioperative Outcomes of Extensive Bowel Resection During Cytoreductive Surgery in Patients With Advanced Ovarian Cancer. J Surg Oncol (2019) 119:1011–5. doi: 10.1002/jso.25403 30737795

[4] Son JH Chang SJ . Extrapelvic Bowel Resection and Anastomosis in Cytoreductive Surgery for Ovarian Cancer. Gland Surg (2021) 10:1207–11. doi: 10.21037/gs-2019-ursoc-01 PMC8033058 33842266

[5] Nishikimi K Tate S Kato K Matsuoka A Shozu M . Well-Trained Gynecologic Oncologists can Perform Bowel Resection and Upper Abdominal Surgery Safely. J Gynecol Oncol (2020) 31:e3. doi: 10.3802/jgo.2020.31.e3 31788993 PMC6918882

[6] Sato K Horiguchi M Kimura M So E Kojima T Tamuna M . Primary Cytoreductive Surgery of Advanced Ovarian Cancer With Special Reference to the Significance of Bowel Resection. Gan To Kagaku Ryoho (1989) 16:1070–7. 2730012

[7] Gallotta V Fanfani F Vizzielli G Panico G Rossitto C Gagliardi ML . Douglas Peritonectomy Compared to Recto-Sigmoid Resection in Optimally Cytoreduced Advanced Ovarian Cancer Patients: Analysis of Morbidity and Oncological Outcome. Eur J Surg Oncol (2011) 37:1085–92. doi: 10.1016/j.ejso.2011.09.003 21945640

[8] Kim HS Kim EN Jeong SY Chung HH Kim YB Kim JW . Comparison of the Efficacy of Low Anterior Resection With Primary Anastomosis and Hartmann’s Procedure in Advanced Primary or Recurrent Epithelial Ovarian Cancer. Eur J Obstet Gynecol Reprod Biol (2011) 156:194–8. doi: 10.1016/j.ejogrb.2011.01.003 21288627

[9] Mercado C Zingmond D Karlan BY Sekaris E Gross J Maggard-Gibbons M . Quality of Care in Advanced Ovarian Cancer: The Importance of Provider Specialty. Gynecol Oncol (2010) 117:18–22. doi: 10.1016/j.ygyno.2009.12.033 20106512

[10] Qin QY Wu YL Cai YH Kuang YY He YJ Huang XY . Clinical Features and Prognosis of Anastomotic Leak After Anterior Resection for Rectal Cancer Following Neoadjuvant Chemoradiotherapy. Zhonghua Wei Chang Wai Ke Za Zhi (2021) 24:513–22. doi: 10.3760/cma.j.cn.441530-20200601-00330 34148316

[11] Ahmad NZ Abbas MH Khan SU Parvaiz A . A Meta-Analysis of the Role of Diverting Ileostomy After Rectal Cancer Surgery. Int J Colorectal Dis (2021) 36:445–55. doi: 10.1007/s00384-020-03771-z 33064212

[12] Wen R Zheng K Zhang Q Zhou L Liu Q Yu G . Machine Learning-Based Random Forest Predicts Anastomotic Leakage After Anterior Resection for Rectal Cancer. J Gastrointest Oncol (2021) 12:921–32. doi: 10.21037/jgo-20-436 PMC8261311 34295545

[13] Spannenburg L Sanchez Gonzalez M Brooks A Wei S Li X Liang X . Surgical Outcomes of Colonic Stents as a Bridge to Surgery Versus Emergency Surgery for Malignant Colorectal Obstruction: A Systematic Review and Meta-Analysis of High Quality Prospective and Randomised Controlled Trials. Eur J Surg Oncol (2020) 46:1404–14. doi: 10.1016/j.ejso.2020.04.052 32418754

[14] Rutherford C Müller F Faiz N King MT White K . Patient-Reported Outcomes and Experiences From the Perspective of Colorectal Cancer Survivors: Meta-Synthesis of Qualitative Studies. J Patient Rep Outcomes (2020) 4:27. doi: 10.1186/s41687-020-00195-9 32335745 PMC7183519

[15] Stavropoulou A Vlamakis D Kaba E Kalemikerakis I Polikandrioti M Fasoi G . ‘Living With a Stoma’: Exploring the Lived Experience of Patients With Permanent Colostomy. Int J Environ Res Public Health (2021) 18:8512. doi: 10.3390/ijerph18168512 34444262 PMC8393572

[16] Japan Society of Gynecologic Oncology JSGO . The Gynecol Oncol (2020) Japan Society of Gynecologic Oncology Guidelines for the Treatment of Ovarian Cancer, Fallopian Tube Cancer, and Primary Peritoneal Cancer. J Gynecol Oncol (2021) 32(2):e49. doi: 10.3802/jgo.2021.32.e49 33650343 PMC7930451

[17] Gynecological Oncology Committee of Chinese Anti Cancer Association . Guidelines for the Diagnosis and Treatment of Ovarian Malignant Tumors. 2021 Version. China Oncol (2020) 31:490–500. doi: 10.19401/j.cnki.1007-3639.2021.06.07

[18] Canlorbe G Touboul C Chargari C Bentivegna E Maulard A Pautier P . Transitory Stoma at the Time of Complete Cytoreductive Surgery Affects Survival for Patients With Advanced-Stage Ovarian Cancer. Anticancer Res (2018) 38:1517–23. doi: 10.21873/anticanres.12379 29491080

[19] Fleetwood VA Kubasiak JC Janssen I Myers JA Millikan KW Deziel DJ . Primary Anastomosis Versus Ostomy After Colon Resection During Debulking of Ovarian Carcinomatosis: A NSQIP Analysis. Ann Med Surg (2016) 81:302–7. doi: 10.1177/000313481608200413 27097621

[20] Lago V Sanchez-Migallón A Flor B Padilla-Iserte P Matute L García-Granero ÁVerifytat . Comparative Study of Three Different Managements After Colorectal Anastomosis in Ovarian Cancer: Conservative Management, Diverting Ileostomy, and Ghost Ileostomy. Int J Gynecol Cancer (2019) 29:1170–6. doi: 10.1136/ijgc-2019-000538 31296558

[21] Grimm C Harter P Alesina PF Prader S Schneider S Ataseven B . The Impact of Type and Number of Bowel Resections on Anastomotic Leakage Risk in Advanced Ovarian Cancer Surgery. Gynecol Oncol (2017) 146:498–503. doi: 10.1016/j.ygyno.2017.06.007 28610745

[22] Lago V Fotopoulou C Chiantera V Minig L Gil-Moreno A Cascales-Campos PA . Risk Factors for Anastomotic Leakage After Colorectal Resection in Ovarian Cancer Surgery: A Multi-Centre Study. Gynecol Oncol (2019) 153:549–54. doi: 10.1016/j.ygyno.2019.03.241 30952369

[23] Mazina V Wang TN Gray HJ . Modifying Risk Factors for Anastomotic Leak in Gynecologic Oncology Surgery. Gynecol Oncol (2020) 159:2–78. doi: 10.1016/j.ygyno.2020.06.105

[24] Fournier M Huchon C Ngo C Bensaid C Bats AS Combe P . Morbidity of Rectosigmoid Resection in Cytoreductive Surgery for Ovarian Cancer. Risk Factor Analysis Eur J Surg Oncol (2018) 44:750–3. doi: 10.1016/j.ejso.2018.01.005 29580734

[25] Gallotta V Fanfani F Fagotti A Chiantera V Legge F Gueli Alletti S . Mesenteric Lymph Node Involvement in Advanced Ovarian Cancer Patients Undergoing Rectosigmoid Resection: Prognostic Role and Clinical Considerations. Ann Surg Oncol (2014) 21:2369–75. doi: 10.1245/s10434-014-3558-0 24558070

[26] Kalogera E Nitschmann CC Dowdy SC Cliby WA Langstraat CL . A Prospective Algorithm to Reduce Anastomotic Leaks After Rectosigmoid Resection for Gynecologic Malignancies. Gynecol Oncol (2017) 144:343–7. doi: 10.1016/j.ygyno.2016.11.032 27919575

[27] Gockley AA Fiascone S Hicks Courant K Pepin K Del Carmen M Clark RM . Clinical Characteristics and Outcomes After Bowel Surgery and Ostomy Formation at the Time of Debulking Surgery for Advanced-Stage Epithelial Ovarian Carcinoma. Int J Gynecol Cancer (2019) 29:585–92. doi: 10.1136/ijgc-2018-000154 30833444

[28] Lago V Fotopoulou C Chiantera V Minig L Gil-Moreno A Cascales-Campos PA . Indications and Practice of Diverting Ileostomy After Colorectal Resection and Anastomosis in Ovarian Cancer Cytoreduction. Gynecol Oncol (2020) 158:603–7. doi: 10.1016/j.ygyno.2020.05.047 32571682

[29] Tseng JH Suidan RS Zivanovic O Gardner GJ Sonoda Y Levine DA . Diverting Ileostomy During Primary Debulking Surgery for Ovarian Cancer: Associated Factors and Postoperative Outcomes. Gynecol Oncol (2016) 142:217–24. doi: 10.1016/j.ygyno.2016.05.035 PMC4961543 27261325

[30] Koscielny A Ko A Egger EK Kuhn W Kalff JC Keyver-Paik MD . Prevention of Anastomotic Leakage in Ovarian Cancer Debulking Surgery and its Impact on Overall Survival. Anticancer Res (2019) 39:5209–18. doi: 10.21873/anticanres.13718 31519635

[31] Bartl T Schwameis R Stift A Bachleitner-Hofmann T Reinthaller A Grimm C . Predictive and Prognostic Implication of Bowel Resections During Primary Cytoreductive Surgery in Advanced Epithelial Ovarian Cancer. Int J Gynecol Cancer (2018) 28:1664–71. doi: 10.1097/IGC.0000000000001369 30371563

[32] Tozzi R Casarin J Baysal A Pinelli C Matak L Ghanbarzadeh N . Morbidity of Multiple Bowel Resection Compared to Single Bowel Resection After Debulking Surgery for Ovarian Cancer. Eur J Obstet Gynecol Reprod Biol (2019) 240:215–9. doi: 10.1016/j.ejogrb.2019.07.011 31326636

[33] Bertelsen VM Ørtoft G Petersen LK . Bowel Resection With Either Stoma or Anastomosis in Treatment of Advanced Ovarian Cancer. Int J Gynecol Cancer 26:800. doi: 10.1097/01.IGC.0000503327.50238.5c

[34] Lucas E Flor B Ponce M Frasson M Pous S Carvajal N . Poster Abstracts. Colorectal Dis (2016) 18:44–125. doi: 10.1111/codi.13445

[35] Lindner S von Rudno K Gawlitza J Hardt J Sandra-Petrescu F Seyfried S . Flexible Endoscopy is Enough Diagnostic Prior to Loop Ileostomy Reversal. Int J Colorectal Dis (2021) 36:413–7. doi: 10.1007/s00384-020-03766-w PMC7801265 33048240

[36] Blas FL Luis SG Gianluca P Matteo F Álvaro GG Marta P . ‘Virtual Ileostomy’ Combined With Early Endoscopy to Avoid a Diversion Ileostomy in Low or Ultralow Colorectal Anastomoses. A Preliminary Report. Langenbecks Arch Surg Blas (2019) 404:375–83. doi: 10.1007/s00423-019-01776-z 30919049

[37] Kim S Kim MH Oh JH Jeong SY Park KJ Oh HK . Predictors of Permanent Stoma Creation in Patients With Mid or Low Rectal Cancer: Results of a Multicentre Cohort Study With Preoperative Evaluation of Anal Function. Colorectal Dis (2020) 22:399–407. doi: 10.1111/codi.14898 31698537

